# Simple integrative preprocessing preserves what is shared in data sources

**DOI:** 10.1186/1471-2105-9-111

**Published:** 2008-02-21

**Authors:** Abhishek Tripathi, Arto Klami, Samuel Kaski

**Affiliations:** 1 Department of Computer Science, P.O. Box 68, FI-00014, University of Helsinki, Finland; 2 Helsinki Institute for Information Technology, Finland; 3 Department of Information and Computer Science, Helsinki University of Technology, P.O. Box 5400, FI-02015 HUT, Finland

## Abstract

**Background:**

Bioinformatics data analysis toolbox needs general-purpose, fast and easily interpretable preprocessing tools that perform data integration during exploratory data analysis. Our focus is on vector-valued data sources, each consisting of measurements of the same entity but on different variables, and on tasks where source-specific variation is considered noisy or not interesting. Principal components analysis of all sources combined together is an obvious choice if it is not important to distinguish between data source-specific and shared variation. Canonical Correlation Analysis (CCA) focuses on mutual dependencies and discards source-specific "noise" but it produces a separate set of components for each source.

**Results:**

It turns out that components given by CCA can be combined easily to produce a linear and hence fast and easily interpretable feature extraction method. The method fuses together several sources, such that the properties they share are preserved. Source-specific variation is discarded as uninteresting. We give the details and implement them in a software tool. The method is demonstrated on gene expression measurements in three case studies: classification of cell cycle regulated genes in yeast, identification of differentially expressed genes in leukemia, and defining stress response in yeast. The software package is available at .

**Conclusion:**

We introduced a method for the task of data fusion for exploratory data analysis, when statistical dependencies between the sources and not within a source are interesting. The method uses canonical correlation analysis in a new way for dimensionality reduction, and inherits its good properties of being simple, fast, and easily interpretable as a linear projection.

## Background

Combining evidence from several heterogeneous data sources is a central operation in computational systems biology. We assume several vector-valued data sources, such that each source consists of measurements from the same object or entity, but on different variables.

In modeling in general, when it is possible to make sufficiently detailed modeling assumptions, data integration is in principle straightforward. Given a statistical model of how transcriptional regulation works, for instance, the Bayesian framework tells how to integrate gene expression data, prior knowledge, and transcription factor finding data. Lots of practical problems of course remain to be solved. Alternatively, in a classification task of proteins to ribosomal or membrane proteins, for instance, integration is likewise straightforward: do the integration such that the classification accuracy is maximized. This has been done effectively in semidefinite programming for kernel methods [1] and using Gaussian Process prior within the Bayesian framework [2].

In exploratory analysis, that is, when "looking at the data" to start data analysis while the hypotheses are still vague, it is not as straightforward to decide how data sources should be integrated. The task of exploring data is particularly important for the current high-throughput data sources, to be able to spot measurement errors and obvious deviations from what was expected of the data, and to construct hypotheses about the nature of the data. Nowadays in bioinformatics applications this stage is typically done using dimensionality reduction and information visualization methods, and clusterings. A good exploratory analysis method is (i) fast to apply interactively, (ii) easily interpretable by the analyst, and (iii) widely applicable. Linear projection methods, as such or as preprocessing for clusterings and other methods, fulfill all these criteria.

Fusing the sources is not trivial since we need to choose from three very different options. If all sources are equally important and there is not special reason to do otherwise, it makes sense to simply concatenate the variables from all sources together, and then continue with the resulting single source. The classical linear preprocessing method for this case is Principal Component Analysis (PCA). The second option is suitable when one of the sources, such as the class indicator in functional classification tasks, is known to be of the most interest. Then it is best to include only those variables or features within each source that are informative of the class variable. A classical linear method applicable in this case is linear discriminant analysis. This second option is supervised, and only applicable when the class information is available.

The third option is to include only those aspects of each source that are *mutually *informative of each other. Those are the shared aspects, and this task can be motivated through two interrelated lines of thought. The first is noise reduction. If the sources are measurements of the same entity corrupted by independent noise, then discarding the source-specific aspects will discard the noise. The second line of motivation is more abstract, to analyze what is interesting in the data. Here the different measurement sources can convey very different kinds of information of the entities being studied. One example is copy number aberrations and expression measurements of the same genes in cancer studies [3], and another is the activation profiles of the same yeast gene in several stressful treatments in the task of defining yeast stress response [4]. In these examples it is what is in common in the sources that we are really interested in. Note that the "noise" may be very structured; its effective definition is that it is source-specific.

Commonalities in data sources have been studied by methods that search for statistical dependencies between them. The earliest method was classical linear Canonical Correlation Analysis (CCA) [5], which has later been extended to nonlinear variants and more general methods that maximize mutual information instead of correlation. Yet, being fast, simple and easily understandable, the linear CCA still has a special place in the data analysis toolbox, analogously to the linear Principal Component Analysis which is still being used heavily instead of all modern dimensionality reduction and factor analysis techniques.

CCA addresses the right problem, searching for commonalities in the data sources. Moreover, being based on eigenvalue analysis it is fast and its results are interpretable as linear correlated components. It is not directly usable as a data fusion tool, however, since it produces separate components and hence separate preprocessing for each source. If the separate outputs could be combined in a way that is both intuitively interpretable and rigorous, the resulting method could become a widely applicable dimensionality reduction tool, analogously to PCA for a single source. Performing dimensionality reduction helps in avoiding overfitting, focusing on the most important effects, and reduces computational cost of subsequent analysis tasks.

In this paper we turn CCA into a data fusion tool by showing that the justified way of combining the sources is simply to sum together the corresponding CCA components from each source. An alternative view to this procedure is that it is equivalent to whitening each data source separately, and then running standard PCA on their combination. This is one of the standard ways of computing CCA, but for CCA the eigenvectors are finally split into parts corresponding to the sources. So the connection to CCA is almost trivial and it is amazing that, as far as we know, it has not been utilized earlier in this way.

Our contribution in this paper is to point out that CCA can be used to build a general-purpose preprocessing or feature extraction method, which is fast, and easily interpretable. There are two alternative interpretations. The first is the connection to CCA discussed above. The second is that it extends the standard practice of standardizing the mean and variance of each variable separately before dimensionality reduction. Now each data source is standardized instead of each variable.

We have developed a practical software tool for R that incorporates the subtle but crucial choices that need to be made to choose the dimensionality of the solution. The method is demonstrated on three collections of gene expression measurements.

A kernelized version of CCA (KCCA) has been used in specific data fusion tasks (see e.g. [6,7]) and it could be easily extended to be used in the same way as the linear CCA here. We will focus on the linear mappings for two practical reasons: Computation of the linear version is fast and the components are more easily interpretable. In particular, the kernelized version does not reveal which of the original features cause the dependencies between sources.

## Results and Discussion

### Algorithm

In this section we first explain a simple two-step procedure, based on whitening and PCA, for finding the aspects shared by the sources, and then show how the same fusion solution can equivalently be derived from the result of applying a generalized CCA to the collection. The two-step procedure provides the intuition for the approach: First *remove *the within-data variation, and then *capture *all the variation that is still remaining. The connection to CCA then demonstrates how the procedure provides a solution to the issue of combining the separate components CCA gives.

Denote a collection of *p *data sets by {**X**_1_,...,**X**_*p*_}, where each **X**_*i *_is a *m *× *n*_*i *_matrix such that *m *≫ *N*, and *N *= ∑*n*_*i*_. The rows of the matrices correspond to the same object in each set, while the columns correspond to features that need not be the same in the data sets. For example, in traditional expression analyses the rows would be genes and the columns would be conditions, treatments, time points, etc. For notational simplicity, we assume zero mean data.

In the first step, each data set is whitened to remove all within-data correlations, and the data are scaled so that all dimensions have equal variance. The whitened version ${\bar{X}}_{i}$ of a data matrix **X**_*i *_is given by ${\bar{X}}_{i}=X_{i}W_{X_{i}}$, where $W_{X_{i}}$ is the whitening matrix. The whitening matrix is simply $W_{X_{i}}=C_{X_{i}}^{-1/2}$, where $C_{X_{i}}$ is the covariance matrix of **X**_*i*_.

After each data set has been whitened, the next step is to find the shared variation in them. This is done by principal component analysis (PCA) on the columnwise concatenated whitened data sets. Since all the within-data structure PCA could extract has been removed, it can only find variation shared by at least two of the data sets, and the maximum variance directions it searches for correspond to the highest between-data correlations.

Formally, applying PCA to the columnwise concatenation of the whitened data sets $Z=[{\bar{X}}_{1},...,{\bar{X}}_{p}]$ yields the factorization

(1) **C**_*Z *_= **V Λ V**^*T*^,

where the orthonormal matrix **V **contains the eigenvectors, **Λ **is a diagonal matrix of projection variances, and **C**_*Z *_is the covariance matrix of **Z**.

Projecting **Z **onto the first *d *eigenvectors **V**_*d *_corresponding to the *d *largest eigenvalues gives the *d *principal components, which are the optimal *d*-dimensional representation in terms of the shared variance. The whole data collection becomes integrated into

(2) **P**_*d *_= **ZV**_*d*_,

where **P**_*d *_is of size *m *× *d *and contains a *d*-dimensional feature vector for each of the analyzed objects. The idea is then simply to use this new representation for any further analysis, which can be made using any method that operates on vectorial data. The whole procedure can be seen as fusing the collection of data sets into a single set, while at the same time reducing the total dimensionality of the data to find the most reliable shared effects.

As mentioned in the *Background *section, the above two-step procedure is equivalent to running CCA on the collection and summing the separate components from each source. The connection is derived here for two data sets. The proof extends easily for several data sets, for one of the many alternative generalizations of CCA.

CCA is a method for finding linear projections of two sets of variables so that the correlation between the projections is maximal. CCA is often formulated as a generalized eigenvalue problem

(3) $$\left(\begin{array}{cc} C_{11} & C_{12}\\ C_{21} & C_{22} \end{array}\right)\left(\begin{array}{c} u_{1}\\ u_{2} \end{array}\right)=\lambda \left(\begin{array}{cc} C_{11} & 0\\ 0 & C_{22} \end{array}\right)\left(\begin{array}{c} u_{1}\\ u_{2} \end{array}\right),$$

where **C**_*ij *_denotes the (cross-)covariance of **X**_*i *_and **X**_*j*_. The eigenvalues *λ *of the solution appear as pairs 1 + *ρ*_1_, 1 -*ρ*_1_,...,1 + *ρ*_*p*_, 1 - *ρ*_*p*_, 1,...,1, where *p *= min(*n*_1_, *n*_2_), and (*ρ*_1_,...,*ρ*_*p*_) are the canonical correlations. The canonical weights corresponding to the canonical correlations are $u^{i}={\left[u_{1,i}^{T},u_{2,i}^{T}\right]}^{T},$, *i *= 1,...,*p*.

In conventional use of CCA we are usually interested in the correlations, the canonical weights **u**^*i*^, and the canonical scores, defined as projections of **X**_1 _and **X**_2 _on the corresponding canonical weights. Next we show how the combined data set (2) can be obtained from the canonical scores, thus providing a way of using CCA to find a single representation that captures the dependencies.

For a single component, (1) can be equivalently written as

$$\left(\begin{array}{cc} {\bar{C}}_{11} & {\bar{C}}_{12}\\ {\bar{C}}_{21} & {\bar{C}}_{22} \end{array}\right)v=\alpha v,$$

where *α *is the variance, **v **is the corresponding principal component, and ${\bar{C}}_{ij}$ denotes the (cross-)covariance of ${\bar{X}}_{i}$ and ${\bar{X}}_{j}$. Due to the whitening, the ${\bar{C}}_{11}$ and ${\bar{C}}_{22}$ are identity matrices. We can alternatively write ${\bar{C}}_{12}=W_{1}^{T}C_{12}W_{2}$ and ${\bar{C}}_{21}=W_{2}^{T}C_{21}W_{1}$, leading to

$$\left(\begin{array}{cc} 0 & W_{1}^{T}C_{12}W_{2}\\ W_{2}^{T}C_{21}W_{1} & 0 \end{array}\right)v=(\alpha -1)v,$$

where **Iv **has been subtracted from both sides. Equivalently,

(4) $$\left(\begin{array}{cc} 0 & C_{12}\\ C_{21} & 0 \end{array}\right)\left(\begin{array}{cc} W_{1} & 0\\ 0 & W_{2} \end{array}\right)v=(\alpha -1){\left(\begin{array}{cc} W_{1}^{T} & 0\\ 0 & W_{2}^{T} \end{array}\right)}^{-1}v.$$

Let us denote $\left(\begin{array}{cc} W_{1} & 0\\ 0 & W_{2} \end{array}\right)$ by diag [**W**_1_, **W**_2_], and multiply the right hand side of (4) by the identity matrix **I **= diag[**W**_1_, **W**_2_]^-1^diag [**W**_1_, **W**_2_]. On the right side of the equation we then have the term diag[**W**_1_^*T*^, **W**_2_^*T*^]^-1 ^diag[**W**_1_, **W**_2_]^-1 ^= diag [**C**_11_, **C**_22_] based on the definition of the whitening matrix, and thus (4) can be written as

(5) $$\left(\begin{array}{cc} 0 & C_{12}\\ C_{21} & 0 \end{array}\right)\hat{v}=(\alpha -1)\left(\begin{array}{cc} C_{11} & 0\\ 0 & C_{22} \end{array}\right)\hat{v},$$

where $\hat{v}=\text{diag}[W_{1},W_{2}]v$. Adding $I\hat{v}$ to both sides gives equation structurally identical to (3). Both methods thus lead to the same eigenvalues, i.e. *λ *= *α*, and the eigenvectors are related by a linear transformation,

$$\text{diag}[W_{1},W_{2}]v={\left[u_{1}^{T},u_{2}^{T}\right]}^{T}.$$

The combined representation (2) of *d *dimensions can be written in terms of canonical scores as **P**_*d *_= **ZV**_*d *_= [**X**_1_, **X**_2_]diag[**W**_1_, **W**_2_]**V**_*d *_= [**X**_1_, **X**_2_] ${\left[U_{1,d}^{T},U_{2,d}^{T}\right]}^{T}$ = **X**_1_**U**_1,*d *_+ **X**_2_**U**_2,*d*_, where **U**_1,*d *_and **U**_2,*d *_are the first *d *canonical directions of the two data sets.

CCA can be generalized to more than two data sets in several ways [8], and the two-step procedure described here is equivalent to the one formulated as solving a generalized eigenvector problem **Cu **= *λ***Du**, where **C **is the covariance matrix of the column-wise concatenation of the **X**_*i *_and **D **is a block-diagonal matrix having the dataset-specific covariance matrices **C**_*ii *_on its diagonal. Here **u **is a row-wise concatenation of the canonical weights corresponding to the different data sets. The proof follows along the same lines as for two data sets, and again the combined data set for any $d<\sum_{i=1}^{p}n_{i}$ dimensions can be written in terms of the generalized CCA results as

$$P_{d}=\sum_{i=1}^{p}X_{i}U_{i,d},$$

where each **U**_*i,d *_contains the *d *eigenvectors corresponding to the *d *largest eigenvalues.

In summary, the simple linear preprocessing method of whitening followed by PCA equals computing the generalized CCA on a collection of data sets and summing the canonical scores of the data sets. In practice it does not matter in which way the result is obtained, but the two-step procedure illustrates more clearly why this kind of approach is useful for data integration. Furthermore, it is not limited to linear projections, and the same motivation could be extended to different kind of models. In practice implementing the first step might, however, be difficult in more complex models.

#### Choice of dimensionality

The dimensionality of the projection can be chosen to be fixed, such as two or three for visualization, or alternatively an "optimal" dimensionality can be sought. In this section we introduce our suggestion for optimizing the dimensionality. Intuitively, the dimensionality should be high enough to preserve most of the shared variation and yet low enough to avoid overfitting. The first few components contain most of the reliable shared variation among the data sets, while the last components may actually represent just noise, and thus dropping some of the dimensions makes the method more robust.

The maximum dimensionality is the sum of the dimensionalities of the data sets, but in practice already a considerably smaller dimensionality is often sufficient, and in fact leads to a better representation due to suppression of noise. Note also that in the case of two data sets the number of unique projections is only the minimum of the data dimensionalities.

In a nutshell, we increase the dimensionality one at a time, testing with a randomization test that the new dimension captures shared variation. To protect from overfitting, all estimates of captured variation will be computed using a validation set, i.e., for data that has not been used when computing the components (dimensions). The randomization test essentially compares the shared variance along the new dimension to the shared variance we would get under the null-hypothesis of mutual independency. When the shared variance does not differ significantly from the null-hypothesis, the final dimensionality has been reached.

To compute the shared variance of the original data, we divide the data into training, $X_{i}^{t}$ and validation data, $X_{i}^{v}$. The two step procedure described in the *Algorithm *subsection is applied to the training data to compute the eigenvectors **V**^*t *^and the whitening matrix **W**^*t*^, where **W**^*t *^is a block diagonal matrix containing the whitening matrices for each matrix in training data. The fused representation for the validation data is computed as $P_{d}^{v}=X^{v}W^{t}V_{d}^{t}$, where **X**^*v *^is the columnwise concatenation of the validation data matrices. Variance in the fused representation is now our estimate of shared variance. We average the estimate over 3 different splits into training and validation sets.

To compute the shared variance under the null hypothesis, random data sets are created from the multivariate normal distribution with a diagonal covariance matrix where the values in diagonal equal the columnwise variances of $X_{i}^{t}$. The shared variance for the random data is computed in the same way as described above. We repeat the process for 50 randomly created data sets.

The shared variance in the original data is then compared to the distribution of shared variances under the null hypothesis, starting from the first dimension. When the dimensions no longer differ significantly (we used 2% confidence level), we have arrived at the "optimal" dimensionality and the rest of the dimensions are discarded.

Note that assuming normally distributed data in the null hypothesis is consistent with the assumptions implicitly made by CCA. The underlying task is to capture all statistical dependencies in the new representation, and finding correlations (as done by CCA) is equivalent to that only for data from the normal distribution. For considerably non-normal data the choice of dimensionality may not be optimal, but neither is the method itself. Therefore transforming the data so that it would roughly follow normal distribution (such as taking logarithm of gene expression values) would be advisable.

### Implementation

We have implemented the method, including the choice of dimensionality and the validation measures presented in the section *Validation measures*, as an open-source package for R [See Additional file 1].

### Experiments

#### Validation on gene expression data

We first validate the method on three gene expression data sets (described in Section *Methods*), by checking how well it preserves the shared variation in data sets and discards the data-specific variation.

**Figure 1:**
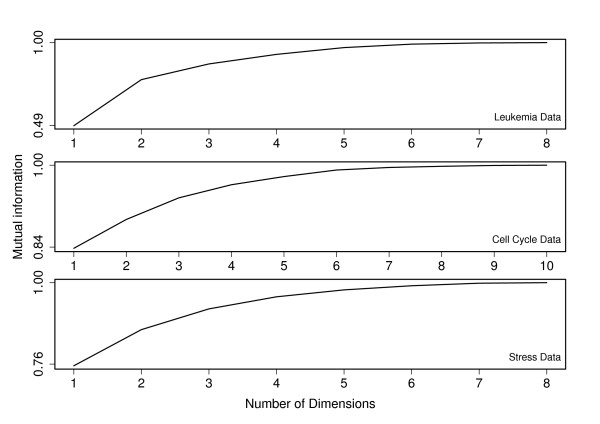
**Mutual Information**. Mutual information for two data sets as a function of the reduced dimensionality. Each subgraph represents mutual information curve for two data sets corresponding to each data collection. The curves for other pairs in each data collection show a similar pattern.

In case of two data sets an estimate of mutual information can be computed directly from the canonical correlations as

$$I(X_{1}U_{1,d},X_{2}U_{2,d})=-\frac{1}{2}\sum_{i=1}^{d}\log(1-\rho_{i}^{2}),$$

based on the assumption of normally distributed data. Consequently we started by confining to pairs of data sources. Figure 1 shows the results for one of the pairs in each collection; the rest are analogous. It is evident that the method retains the shared variation between data sets and the shared variation increases with increasing number of dimensions in the combined data.

For more than two variables, the measures explained in the *Methods *Section are used. We compare the results with PCA of the concatenated data matrices. PCA is equally fast, linear, and unsupervised. Note that the proposed CCA-based method is also unsupervised as no class information is used. Furthermore, since both methods have a global optimum, differences in performance cannot be due to optimization issues. The only difference then is related to the main topic of this paper: whether to model all information in the whole data collection, as PCA does, or only the mutual dependencies.

Shared variance (6) and data-specific (7) variance captured by the fused data were computed for each of the three data collections. The presented results are averages over five-fold cross-validation, and the variances have always been computed for the left-out data. In addition to the PCA comparison, we provide baseline results obtained with random orthonormal projections that have uniform distribution on the unit sphere.

**Figure 2:**
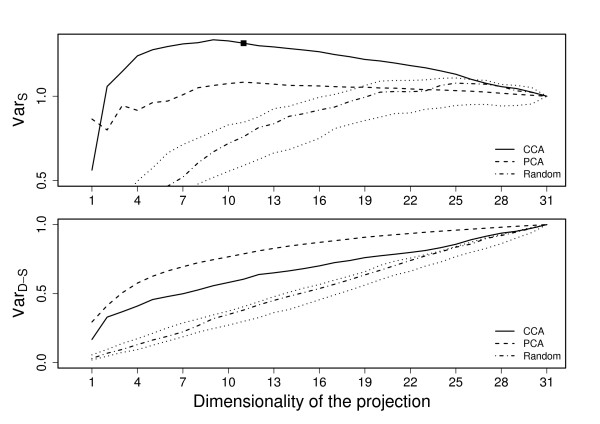
**Shared and Data-specific variation for leukemia data**. Shared (top) and data-specific (bottom) variation retained with CCA (solid line) and PCA (dashed line) as a function of the reduced dimensionality for the leukemia data. The values obtained by random projections (dash-dotted line and dotted confidence intervals) have been included for reference. The suggested dimensionality for the CCA-projection is marked with a tick.

**Figure 3:**
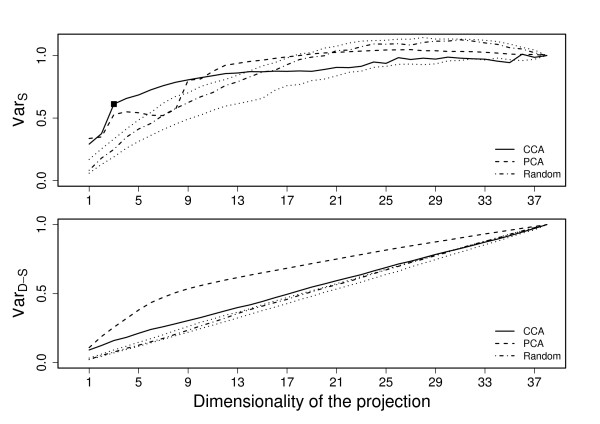
**Shared and Data-specific variation for cell-cycle data**. Shared (top) and data-specific (bottom) variation retained with CCA (solid line) and PCA (dashed line) as a function of reduced dimensionality for the cell-cycle data. The values obtained by random projections (dash-dotted line and dotted confidence intervals) have been included for reference. The suggested dimensionality for the CCA-projection is marked with a tick.

**Figure 4:**
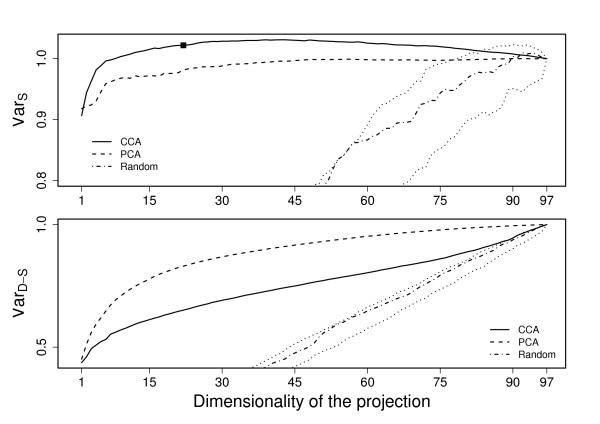
**Shared and Data-specific variation for stress data**. Shared (top) and data-specific (bottom) variation retained with CCA (solid line) and PCA (dashed line) as a function of the reduced dimensionality for the stress data. The values obtained by random projections (dash-dotted line and dotted confidence intervals) have been included for reference. The suggested dimensionality for the CCA-projection is marked with a tick.

The results are presented for each of the data sets in Figures 2, 3, and 4. In all cases it is easily seen that the proposed method retains clearly less data-specific variation than PCA (bottom subfigures), regardless of the dimension. The CCA-based method still keeps more variation than random projections, indicating that it is not purposefully looking for projection directions that would lose more variation than necessary.

At the same time the proposed method retains more between-data variation (top subfigures) for wide range of dimensionalities in all cases. The difference is particularly clear for the leukemia data (Fig. 2) where the CCA-based approach is considerably better than the PCA. In stress data (Fig. 4) the difference is also clear, but PCA is also very good in comparison to the random baseline. For cell-cycle data (Fig. 3) the differences are smaller, but for dimensionalities between 3 and 9 the CCA-based method is still clearly better.

It is striking that in all three cases the PCA, which simply aims to keep maximal variation, is the best also in terms of the shared variation for dimensionality of one. A one-dimensional projection, however, loses a lot of the variation and is not too interesting as a summary of several data sets. Hence, this finding does not have a lot of practical significance.

One notable observation is that especially for the leukemia data (Fig. 2) the between-data variance of the CCA-method is, for a wide range of dimensionalities, higher than the corresponding value for the original collection. This does not, however, seem to have clear operational meaning but is merely a side-effect of the heuristic measure.

The curves of extracted variance can be contrasted to the suggested dimensionalities (see Section *Choice of dimensionality*), marked with ticks in the plots. For two of the three data sets the suggested dimensionality is very close to the maximum point of the between-data variance curve, and when increasing the dimensionality the result remains relatively constant, or even decreases for the leukemia data. While the amount of data-specific variation still keeps increasing, there is no longer a significant amount of shared variation available, and the chosen dimensionality is thus good in terms of these two measures. For the third data collection, the cell-cycle, the suggested dimensionality is somewhat lower than what is needed for maximally capturing the between-data variation. However, as seen in the next section, the chosen dimensionality is still very good for a practical application, providing the best result in the actual case study.

### Prototypical Applications

In this section we will discuss a few prototypical ways in which the method could be applied. The method is a general-purpose tool for integrating a collection of data sets in such a way that the effects common to several sets are enhanced. After the integration step any analysis method operating on vectorial data can be used. Here some simple methods are used for demonstrational purposes. The applications are demonstrated on the same data sets that were used in the technical validation.

#### Shared effects in leukemia subtypes

Pediatric acute lymphoblastic leukemia (ALL) is a heterogeneous disease with subtypes that differ markedly in their cellular and molecular characteristics as well as their response to therapy and subsequent risk of relapse [9]. Combining the expression measurements of the five different ALL subtypes gives a representation where the genes that have similar (or more exactly, statistically dependent) expression profile in several subtypes are similar. Here we are interested in the genes that are highly (over or under) expressed, and thus study the equivalent of differential expression in the combined data set.

The fusion method was applied to combine the five ALL data sets, resulting in a 11-dimensional representation. After this we can proceed as if we only had one data source. It has a 11-dimensional feature vector for each gene, and we separate the 1% of genes that have the highest distance from the origo, implying highest total contribution to the shared variation. This set of genes is compared to the corresponding set obtained from a 11-dimensional PCA projection of the whole collection. In addition, a baseline result computed from the full concatenation of the original data sets is included.

A functional annotation tool, DAVID (Database for Annotation, Visualization and Integrated Discovery) [10] was used to annotate the gene lists to find the gene ontology (GO) enrichments in the biological processes category. The most enriched GO-terms were the same for both CCA and PCA, which is understandable as we are using two linear projection methods on the same collection. We picked the GO-terms which have p-values (Bonferroni corrected) lower than 0.01, and present the counts of the genes from these categories in Table 1. The notable observation is that the CCA-based method has higher count in all but one category, in which the counts are tied. Both methods thus reveal the same kinds of processes, all related to immune response, but CCA is more accurate and is able to include more genes related to these biological processes in the top 1% genes.

**Table 1:** GO enrichment by CCA and PCA

GO term	PCA	CCA	Baseline
Response to biotic stimulus	53, 2.2E-15	61, 2.7E-19	55, 6.2E-17
Defense response	51, 1.1E-14	58, 7.3E-18	53, 3.4E-16
Immune response	47, 3.8E-13	54, 9.5E-17	48, 3.9E-14
Response to pest, pathogen, parasite	29, 7.0E-09	30, 1.4E-08	26, 1.4E-06
Response to other organism	29, 3.0E-08	30, 6.1E-08	26, 4.8E-06
Response to stimulus	61, 6.4E-07	75, 4.1E-12	62, 2.0E-07
Response to stress	35, 9.5E-06	36, 3.6E-05	31, 1.5E-03
Organismal physiological process	54, 5.7E-05	68, 4.8E-10	55, 2.0E-05
Response to external stimulus	22, 1.8E-04	22, 9.4E-04	19, 1.5E-02
Response to wounding	19, 3.3E-04	20, 2.9E-04	16, 3.8E-02

The enriched gene ontology terms from the biological processes category with p-values (Bonferroni corrected) lower than 0.01. Both CCA and PCA result in the same 10 terms, and here they are sorted according to the *p*-value of the gene list obtained with PCA preprocessing. Each cell lists the count of genes in that term, together with the p-value (Bonferroni corrected). In 9 out of 10 the count is higher for CCA, showing that it is able to capture relevant genes with better accuracy, avoiding outliers. The baseline method shares 8 common GO terms with CCA/PCA, and the two different GO enrichments are Antigen presentation, endogeneous antigen (8, 1.5E-4) and Antigen processing, endogeneous antigen via MHC class I (7, 6.3E-3).

#### Classification of cell cycle regulated genes in yeast

The second prototype application is about cell-cycle regulation using the gene expression of *Saccharomyces cerevisiae *[11]. Expression measurements from 5 different experiments are combined with the proposed method. The combined data is used for the classification of cell-cycle regulated genes.

As the new representation is simply a real-valued vector for each gene, several alternative classifiers are applicable; here K-nearest neighbor (KNN) classifier is selected for demonstrational purposes. We use the cell-cycle regulated genes reported by [11] as the class labels, giving a two-class classification problem: either a gene is or is not cell-cycle regulated.

**Figure 5:**
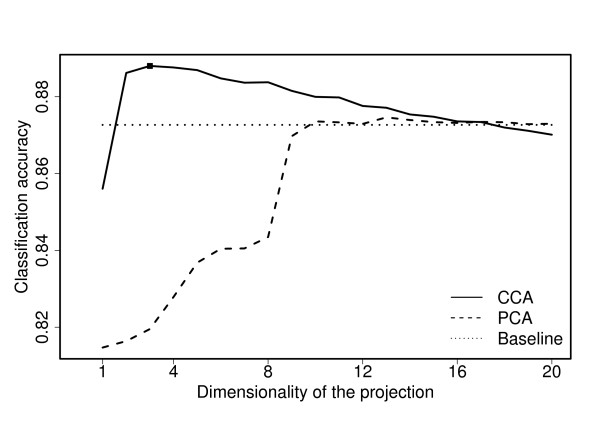
**KNN classification for cell-cycle data**. The classification accuracy obtained using the combined representation as a function of dimensionality. The CCA-based combination (solid line) is clearly superior to PCA-based approach (dashed line) for a wide range of dimensionalities and obtains higher maximal accuracy. As a baseline, the classification accuracy obtained by the concatenation of all original data sets (dotted line) is also included.

The leave-one-out classification accuracy of CCA and PCA projections is shown in Figure 5, together with a baseline obtained by using the full concatenation of the original data sets. It is evident that the CCA-based method provides a considerably better representation for separating the cell-cycle regulated genes from the rest. Already the one-dimensional CCA-projection gives a higher accuracy than what is obtained with an eight-dimensional PCA-projection, and the maximal accuracy is clearly higher for CCA and obtained already with a three-dimensional representation. This is exactly the dimensionality suggested by the procedure explained in Section *Choice of dimensionality*.

#### Defining the environmental stress in yeast

We also study yeast gene expression data from [12,13], consisting of time series of gene expression measurements of *Saccharomyces cerevisiae *in various stressful treatments. The data is combined to study the genes related to general environmental stress response (ESR).

In [12] the environmental stress response of yeast was studied based on a broad collection of different treatments, out of which 9 are used in our experiment. The original analysis relied primarily on a hierarchical clustering of the whole collection, and was thus based on the overall similarity of the expression patterns. While it is able to cluster the genes into sensible categories, it is ideologically comparable to the PCA approach for preprocessing: It does not take into account that not all variation is equally important.

We suggest that it might be a better idea to focus on the variation shared by the different data sources, instead of trying to characterize the similarity based on all variation. Treatment-specific effects would be specific stress responses and if the task is to find a general response, its fingerprint is in the shared variation. Thus the analysis of environmental stress response should start with a preprocessing step like the one suggested here. We demonstrate how the results of such approach differ from those obtained by [12].

**Figure 6:**
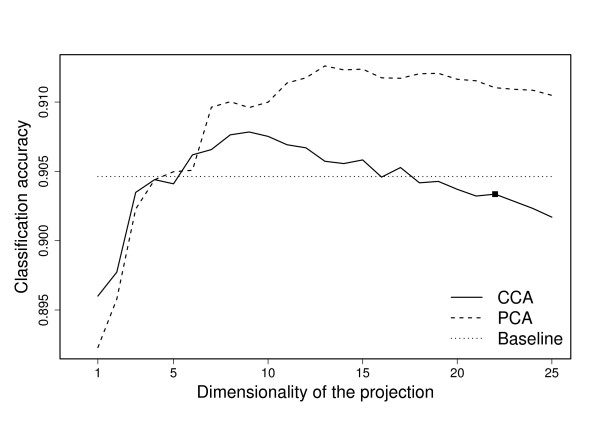
**KNN classification for stress data**. The classification accuracy obtained using the combined representation as a function of dimensionality. The CCA-based combination (solid line) is consistently worse than the PCA-based approach (dashed line), implying that the class labels might not correlate that well with the true shared response. As a baseline, the classification accuracy obtained by the concatenation of all original data sets (dotted line) is also included.

We applied a KNN classifier to the combined data space to classify the genes to belong to the three categories labeled in [12] (a gene is either up- or down-regulated ESR gene, or is not coordinately regulated in stress). The accuracies of CCA and PCA approaches in this task are presented in Figure 6. Again a baseline obtained by using the full concatenation of the original data sets in included. Though the accuracies are similar for some initial dimensionalities, we notice that the accuracy after preprocessing by PCA is higher, by a margin of roughly 0.5% to 1%, for a wide range of dimensionalities including the suggested dimensionality of combined representation, 22, obtained with the method of Section *Choice of dimensionality*. Also, for the higher dimensionalities the baseline method which simply uses the original data is better. As argued above, this does not tell that CCA was the worse preprocessing method, but instead suggests that the original classes have indeed been constructed based on all variation in the data, including treatment-specific responses. This is not desirable since the definition of an ESR gene is that it would be responsive to stress in general. As the data set has slightly less than 6000 genes this corresponds to a difference of roughly 30 to 60 misclassifications. This characterizes the scale of the disagreement between the two fundamentally different approaches to the preprocessing phase.

This result hints that the definitions created after CCA-based preprocessing would be mostly the same as the ones given in [12], but for some roughly 5 – 10% of genes the classification should be changed.

## Conclusion

We studied the problem of data fusion for exploratory data analysis in a setting where the sensible fusion criterion is to look for statistical dependencies between data sets of co-occurring measurements. We showed how a simple summation of the results of a classical method of canonical correlation analysis gives a representation that captures the dependencies, leading to an efficient and robust linear method for the fusion task. It does not solve the data integration task in general, but it shows that the criterion in the data fusion task should not necessarily be to keep all the possible information present in the data collection. Instead, we may want to focus on the aspects shared by different views. We showed how that can be achieved with simple and easily applicable methods.

We demonstrated the validity of the method on three different real gene expression data sets using technical criteria. We further presented three examples on how the method could be used as the preprocessing step in different kinds of analysis tasks.

## Methods

### Data

#### Leukemia data

We used the data from [9]. A subset of the data for each leukemia subtype, BCR-ABL, E2A-PBX1, MLL, TEL-AML1 and T-ALL, was chosen such that the patients in each subtype are homogeneous. A set of hyperdiploid samples was used as a control.

We used RMA (Robust Multi-array Analysis) to preprocess the data, and subtracted the mean of the hyperdiploid samples. In total we analyzed 22,283 genes for 31 patients, divided into 5 data sets.

#### Cell-cycle data

We used the cell-cycle data from [11]. It includes 3 different time courses and 2 different induction experiments. In the original work the data was used to label a set of 800 genes as potentially cell-cycle regulated genes, based on similarity with 104 experimentally verified cell-cycle regulated genes.

We preprocessed the data by imputing missing values with the K-nearest neighbor method, using *K *= 10. After that the data was Fourier-transformed, and the power spectrum was used for the analysis. In the end we had 5,670 genes, including 724 out of 800 cell-cycle regulated genes defined in [11]. The total number of features in the 5 data sets was 38.

#### Yeast stress data

We used the yeast gene expression data under various stress conditions from [12,13]. We picked 15 different conditions, 9 from [12] and 6 from [13], resulting in 97 dimensions in total. We then combined them in order to study genes related to general environmental stress response (ESR).

We normalized all time series with their respective zero-points, and imputed missing values by gene-wise averages within each data set. After combining the genes from both sources we got 5,998 genes, out of which 868 were identified as ESR-genes by [12].

### Validation measures

The method aims to keep all the variance that is shared among the data sets, while ignoring the variation that is specific to only one of them. In this section we introduce measures on how well this is achieved in real applications. Since there is not straightforward way of quantifying the degree of dependency for several high-dimensional data sources (correlation is only defined for two variables, and estimation of multivariate generalizations of mutual information is difficult), we used two partly heuristic variance-based criteria as comparison measures.

Both measures are based on examining reconstructions of the original data sets. If an integrated representation **P**_*d *_of full dimensionality is used then it is naturally possible to create a perfect reconstruction, but lower dimensionality introduces errors. We want to measure to what degree the preserved information was shared and to what degree specific to individual data sets.

The reconstruction ${\hat{X}}_{i}$ of the *i*th data set is obtained by extracting the columns corresponding to the *i*th data set from $\hat{Z}=P_{d}V_{d}^{†}$ and multiplying that by the inverse of the whitening matrix **W**_*i*_. Here $V_{d}^{†}$ is the generalized inverse of **V**_*d*_, defined as $V_{d}^{†}={\left(V_{d}^{T}V_{d}\right)}^{-1}V_{d}^{T}$.

The first criterion measuring the data-specific variation after the dimensionality reduction to *d *dimensions is defined as

(6) $${\text{Var}}_{\text{S}}=\sum_{i=1}^{p}\text{Trace}(C_{{\hat{X}}_{i}}).$$

Each term in the sum is simply the variance of a single reconstruction, and the sum matches the total variation in the collection of data sets. The measure is further normalized so that the value for *d *= *N*, the full dimensionality, is one.

For the shared variation we measure the pairwise variation between all pairs of data sets. The measure uses the same reconstructed data sets, and is defined as

(7) $${\text{Var}}_{\text{D-S}}=\sum_{i=1}^{p-1}\sum_{j=i+1}^{p}\text{Trace}({\hat{X}}_{i}^{T}{\hat{X}}_{j}),$$

again normalized so that the full dimensionality gives the value one. It is worth noticing that the sum of pairwise variations is not a perfect measure for the shared variation for collections with more than two data sets, but it is computationally simple and intuitive.

## Availability and requirements

Project name: drCCA;

Project home page: ;

Operating system(s): Platform independent;

Programming language: R

License: GNU LGPL;

Any restrictions to use by non-academics: Read GNU LGPL conditions

## Authors' contributions

AT implemented the software and carried out the experiments. All authors participated in developing the algorithm, designing of the experiments, and writing of the manuscript. All authors read and approved the final manuscript.

## Supplementary Material

Additional file 1 A software package in R. An R implementation of the method including the source codes and documentation of the software. Click here for file
